# Toward explainable AI-empowered cognitive health assessment

**DOI:** 10.3389/fpubh.2023.1024195

**Published:** 2023-03-09

**Authors:** Abdul Rehman Javed, Habib Ullah Khan, Mohammad Kamel Bader Alomari, Muhammad Usman Sarwar, Muhammad Asim, Ahmad S. Almadhor, Muhammad Zahid Khan

**Affiliations:** ^1^Department of Cyber Security, Air University, Islamabad, Pakistan; ^2^Department of Electrical and Computer Engineering, Lebanese American University, Byblos, Lebanon; ^3^Department of Accounting and Information Systems, College of Business and Economics, Qatar University, Doha, Qatar; ^4^Department of Computer Games Development, Air University, Islamabad, Pakistan; ^5^Department of Cyber Security, National University of Computer and Emerging Science, Islamabad, Pakistan; ^6^College of Computer and Information Sciences, Jouf University, Sakakah, Saudi Arabia; ^7^Department of Computer Science & IT, University of Malakand, Chakdara, Pakistan

**Keywords:** explainable AI, advanced sensors, assistive technology, key feature extraction, human activity recognition, Internet of Things, healthcare

## Abstract

Explainable artificial intelligence (XAI) is of paramount importance to various domains, including healthcare, fitness, skill assessment, and personal assistants, to understand and explain the decision-making process of the artificial intelligence (AI) model. Smart homes embedded with smart devices and sensors enabled many context-aware applications to recognize physical activities. This study presents *XAI-HAR*, a novel XAI-empowered human activity recognition (HAR) approach based on key features identified from the data collected from sensors located at different places in a smart home. *XAI-HAR* identifies a set of new features (i.e., the total number of sensors used in a specific activity), as *physical key features selection (PKFS)* based on weighting criteria. Next, it presents *statistical key features selection (SKFS)* (i.e., mean, standard deviation) to handle the outliers and higher class variance. The proposed *XAI-HAR* is evaluated using machine learning models, namely, random forest (RF), K-nearest neighbor (KNN), support vector machine (SVM), decision tree (DT), naive Bayes (NB) and deep learning models such as deep neural network (DNN), convolution neural network (CNN), and CNN-based long short-term memory (CNN-LSTM). Experiments demonstrate the superior performance of *XAI-HAR* using RF classifier over all other machine learning and deep learning models. For explainability, *XAI-HAR* uses Local Interpretable Model Agnostic (LIME) with an RF classifier. *XAI-HAR* achieves 0.96% of F-score for health and dementia classification and 0.95 and 0.97% for activity recognition of dementia and healthy individuals, respectively.

## 1. Introduction

Smart home and artificial intelligence (AI)-based healthcare systems are appreciated as an excellent paradigm to solve privacy issues in smart homes (1–7). Explainable artificial intelligence (XAI) is the explainable category of AI (black box) in which humans can understand the solution results (8). Smart homes support automated sustainability to encourage smart cities, smart communities, and high technology-driven solutions (9, 10). Smart homes provide sustainable health solutions such as supporting cognitively impaired individuals by assessing their daily life routine, remote monitoring of home devices for activity recognition, emotion analysis, and depression estimation (11–17).

Currently, neuropsychologists and clinicians are interested in insight into an individual's functional ability to detect diseases early (18–20). There are many solutions to track and monitor an individual's functional ability, such as wearable sensors, vision-based recognition, Wi-Fi-based activity recognition, smartphone-based human activity recognition (HAR), and intelligent homes utilizing the Internet of Things (IoT) (21–26). To assess the functional ability of cognitively impaired individuals, IoT-oriented smart home infrastructures are the most suitable (27–30). Smart homes play an important role in driving the smart cities' revolution by incorporating IoT that connects several devices, systems, and technologies to achieve health-related tasks. A smart home infrastructure is equipped with robust and autonomous smart sensors, for instance, motion, temperature, pressure, and electricity usage sensors, to provide assisted living solutions (31). Actions done in a smart home includes eating, sleeping, cooking, medications, task support parallelism, sequence, and interruption, such as listening to a phone call and writing cards while cooking. There is much discussion about the validity of using an IoT-oriented smart home infrastructure for smart home residents' functional ability assessment. For example, the work of Stavrotheodoros et al. (23) suggested that daily life functional activity assessment is a reasonable way to measure the decline in perceptions. The authors in Wilson et al. (32) argue that data collection is more subtle in a smart home habitat than in a dedicated laboratory environment.

This study presents an activity recognition approach, *XAI-HAR*, to identify key features from a high dimensional feature matrix and augments statistical features to generalize the process of smart home recognizing activities. It is important to retain better the original meaning and representation of a feature matrix to understand a cognitively impaired individual's functional ability. This study makes the following contributions:

- A novel XAI-empowered HAR assessment approach based on key feature identification from the data collected from smart sensors located at different places of a sustainable smart home.
- Introduced a weighting criterion to the sensor events produced in a smart home.
- Provide a combination of the new feature set based on physical key features selection (PKFS) and statistical key features selection (SKFS) for accurate activity recognition.
- Analyze and validate the effectiveness of both key feature selection approaches on the performance of the recognition of activities using machine learning algorithms.
- *XAI-HAR* effectively enhances the recognition rate with consistent performance.

The rest of the study is organized as follows: The literature review is presented in Section 2. The selected smart home dataset is discussed and presented in Section 3.1. Section 3 details the proposed approach. The experimental setup and results are presented in Section 4. Finally, in Section 5, the conclusion and future work are presented.

## 2. Background

This section presents the related work on the fusion of activity monitoring and XAI.

### 2.1. Activity monitoring

A smart home is embedded with a diversity of smart devices and sensors. A smart home is equipped with temperature, motion, heat, and light sensors that human-specific devices such as smartphones and computers can remotely control. These sensors are intelligent enough to reason about and decide our smart home environment setting (33–35). Recently, IT organizations have offered some frameworks for smart homes in an endeavor to capitalize on the market and facilitate the customers in their service-based smart environment so that the market competition and industrial advancement will return as financial advantages to the general public of the smart urban areas (36).

Recent studies highlight that remote monitoring and assisted living could provide patients with real-time assistance and significantly minimize all risks while performing different daily living actions (29, 30, 37). The authors in Dawadi et al. (28, 30) use smart homes for activity assessment of a resident and reported that it is the optimal way to monitor and assist the patient living in it. The data gathered from the interactive sensors deployed in the surrounding can be utilized to recognize activities of daily living (ADLs) carried out inside a smart home, such as food preparation, drinking water, and medication. ADLs automated recognition is crucial in observing a smart home resident's functional health. According to a survey on assistive technologies, the top priority of caregivers of patients with Alzheimer's disease is to identify and track their activity. In Cook (35), the authors survey a generalized activity model that combines sensor actions from all testbeds into one uniform labeled dataset. They applied three basic machine learning algorithms, such as naive bayes (NB), hidden Markov model (HMM), and conditional random field (CRF), over annotated activities. The research of Sarwar and Javed (38) and Javed et al. (39) is designed to make a helping mechanism that assists individuals to live healthfully. After recognizing the physical activities and consent of guardians, doctors, and intelligent agent rankers, a good healthcare plan is suggested.

The authors in Fong et al. (40) proposed a feature-based mechanism for training classifiers that recognizes human activities. They extracted the spatial features called shadow features, which describe current sensor data positions by modeling the performed activities' momentum. The shadow features also highlight the additional information dimensions for nominating activities in the recognition process. Furthermore, they evaluate the devised approach using a wearable and Kinect-based remote sensor. The authors in Eastwood et al. (41) design a set of physical features representing human motion to augment the statistical features. For activity recognition, the authors in Lu et al. (42) extracted latent features from data acquired from sensors with Beta Process hidden Markov model (43). To do that, first, they used the dependent beta process and later integrated sensors' state constraints into sampling. The trained support vector machine (SVM) recognizes the activities from these latent features.

The approach proposed by Cook (35) aims to learn a generalized activity model by combining sensor events from different age groups, such as younger adults, healthy older adults, older adults with dementia, and pets. They used CRF, NB, and HMM for recognition. To improve activity recognition, a segmental pattern mining approach is proposed, in which the segment is a consecutive time event of the same activity (44). In Dawadi et al. (27), the authors present a study for health assessment of cognitively impaired individuals to track the health status in the early stages of the individuals moving toward the critical stage such as dementia. Their focus was to classify healthy individuals and individuals with dementia.

### 2.2. Explainable artificial intelligence

Reducing healthcare costs and sustaining a healthier life are important driving factors for governments to invest in smart cities. The authors in Chen et al. (45) discuss using machine learning (ML) algorithms to mitigate healthcare anomalies. They propose a 5G-Smart Diabetes system for patients with diabetes using sensors and patient vital analysis. in Eastwood et al. (41) designed a set of physical features representing human motion to augment the statistical features. First, a single-layer feature selection framework is applied to analyze the impact on recognition performance. They analyzed that different feature selection mechanisms extract qualitative features that may, in turn, increase the accuracy of recognition. An analysis is conducted on recognizing activities using quick propagation, Levenberg Marquardt, and batch back propagation algorithms (46). Several features are presented that can be used for activity recognition in Chinellato et al. (47). These features are based on time-related measures (i.e., time of occurrence, duration, and repetition), space-related measures (i.e., location of occurrence, movement), complexity-related measures (i.e., event analysis, person analysis, and object analysis), and inter activity-related measures. They used linear discriminative analysis (LDA), random forest (RF), NB, and SVM for recognition.

In summary, the current studies of feature selection lack in selecting a significant feature subset from the whole dataset as the best representative of all features (48). Some drawbacks of the feature selection methods discussed in the literature (40, 49) are: (1) In the case of a smart home, the location of a sensor can be the best feature to represent the whole feature matrix, but it may not correctly the activities performed at other locations or interleaved locations, (2) A feature considered best for one activity can be worst for some other activities such as the location feature, (3) A feature representing the activities of healthy individuals may not correctly represent the activities performed by individuals with dementia, and (4) A feature consisting of frequencies of corrupt or damaged sensors.

By considering the above analysis, the following research questions (RQ) are presented:

- **RQ1:** How to identify key features from a high dimensional feature matrix and augment statistical features to generalize the process of smart home-based activity recognition and interpret the results using explainable AI methods?
- **RQ2:** How to retain a feature matrix's original meaning and representation to understand a cognitively impaired individual's functional ability?
- **RQ3:** How to define a weighting criterion for the sensor events produced in a smart home?
- **RQ4:** What is the effectiveness of the weighting criterion on feature selection?

## 3. Methodology

**Figure 1:**
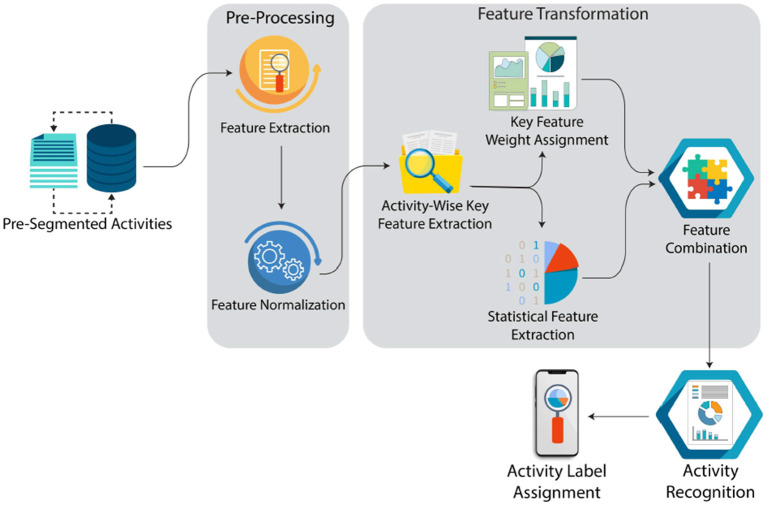
Complete flow of the proposed framework.

In this section, we discuss the suggested approach for activity recognition named *XAI-HAR* for the activities performed by the *healthy* individuals and individuals with *dementia* residing in smart homes. The proposed approach provides a privacy-preserved environment to the resident as the data are collected from motion, pressure, and similar binary state sensors. This approach is being used and recommended by state-of-the-art studies (50–54). *XAI-HAR* consists of two steps: *physical key features selection (PKFS)* and *statistical key features selection (SKFS)* to form a feature matrix corresponding to different well-established contemporary methods used for recognizing activities. *XAI-HAR* presents the concept of selecting vital local features within the dataset. These selected local key features are then transformed for activity recognition. Figure 1 summarizes *XAI-HAR* for data collection and analysis.

### 3.1. Dataset selection

The *XAI-HAR* approach is evaluated the publicly available *Cognitive Assessment Activity (Kyoto)* dataset (27) from the Center for Advanced Studies in Adaptive Systems (CASAS)^1^. The dataset contains passive and automatic sensing data collected from 79 participants from an on-campus smart home testbed at Washington State University. The smart home consists of a living room, kitchen, and dining room on the first floor. The second floor consists of an office, a bathroom, and two bedrooms. The participant's interaction with the smart home is recorded with binary, digital, and analog sensors.

**Figure 2:**
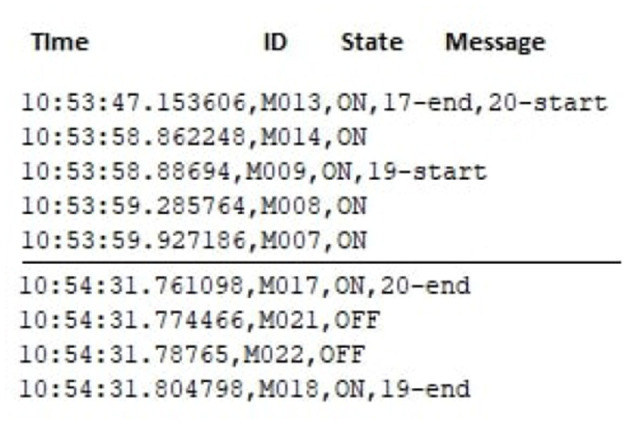
Raw dataset illustration.

Figure 2 provides an overview of the raw dataset. For example, motion sensors (Mxx) are deployed on the ceiling, door sensing devices (Dxx) on cabinets and doors, temperature-sensitive devices (Txx) in each room, light sensors (Lxx), burner sensors (AD1-A), hot water sensors (AD1-B), cold water sensors (AD1-C), whole apartment electricity usage (P001), and item sensors (Ixx) placed on specific items. Sensor events are generated and recorded, whereas the participants perform the activities. Each sensor event comprises a date, time, id, and state (value). Such events are used to make instances for different activities. Sensor events are combined for each activity into a period (starting and ending) as a single sample (instance), representing each participant's activity progress. The sensor events are extracted from the state feature based on the starting and ending activities shown as 19−*start* and 19−*end*. Each sensor event in this activity is counted based on that sensor's triggering and added as an instance in the dataset.

The dataset contains instances of *simple daily life activities*. *Simple daily life activities* are defined as those performed in daily routine and are not interwoven, for instance, taking medicine while doing the dishes. However, in the CASAS dataset, the activities reported by the same sensors and performed in the exact location are difficult to discriminate, such as preparing breakfast, preparing soup, and sweeping the kitchen. Table 1 summarizes the dataset's characteristics used in this study. The ground truth about the personals is generated by comprehensive clinical assessments, which include a review of medical records, neuropsychological testing data, telephone interview of cognitive status (TICS), clinical dementia rating, and some other ways (27).

**Table 1:** Characteristics of the dataset.

**Parameter**	**Value**
Participants	79
Mean Age	66
Healthy	65
Dementia	14
Activities	8
Action 1	Moping the scullery and tidying up the sitting room.
Action 2	Acquiring medicament box along with a dispenser per week, and instruction based fill up of the dispenser.
Action 3	Calligraphy of a birthday card, of address on an envelope, en-wrapping a check.
Action 4	Searching a suitable DVD to listen and watch a news clip.
Action 5	Grabbing a watering can and sprinkling water on each plant in the living area.
Action 6	Replying to a phone call and answering the questions.
Action 7	Cooking soup with the help of the microwave oven.
Action 8	Selection of an appropriate dress from a collection of clothes, for an interview.

### 3.2. Feature extraction

A count of 254 features is retrieved from the sensing data. These features help to identify how well an activity is performed. For example, if a person gets stuck or slows in performing an activity, his/her activity duration time would increase. A participant with dementia would not complete an activity on time due to multiple reasons, such as mistakes wandering and confusion in performing an activity. The following features are those extracted from Dawadi et al. (27):

- Duration: Total time spent to complete an activity.
- Sensor Count: Total number of times a particular sensor is used during the activity.
- Sensor Events: Total number of unique sensor events.
- Activity Completeness: A boolean feature representing the participant's ability to accomplish all eight actions.
- Activity Label: Class label of the individual (i.e., sweeping, watering).

### 3.3. Feature design

Feature design or feature engineering selects the best features and then constructs generic features from the feature matrix capable of efficiently differentiating activities. Feature selection simplifies the model for better understanding and a more straightforward interpretation for users or researchers. A significant benefit of feature selection is that it reduces the number of features the model will train, eventually reducing the training time. In many cases, the feature matrix consists of either dissociated or repeating features that result in overfitting a model, increasing the model's complexity. Usually, the dataset with high dimensions, such as the CASAS dataset which has hundreds of features, may contain a large number of irrelevant and redundant information, which eventually reduces the performance of the learning algorithm (55). Feature selection enhances the model's generalization and accuracy, reducing the chances of overfitting if the right subset of features is selected. To select the dataset's best features, it is necessary to excerpt features set from the raw dataset. The below sections explain two sets of features extracted from the raw dataset.

### 3.4. Physical key feature selection

Physical features are interpreted by human activities performed in a smart home. To systematically identify and assess the usefulness of the most important features for correctly categorizing various activities, many sophisticated techniques can be used to search the compact feature subsets from the dataset. The below equations present the complete process of selecting optimal features from the entire dataset for a smart home resident's cognitive health assessment.

To select the features for *PKFS*, the CASAS dataset (27) is considered well known for cognitive impaired classification. It consists of different activity classes and several activity instances where *D* = *D*_1_, *D*_2_, …, *D*_*x*_ represent the different classes and *I* = *i*_1_, *i*_2_, …, *i*_*k*_*x* represent the instance belonging to each class *D*_*x*_, and features of dataset *D* are the unique sensors *S* = *s*_1_, *s*_2_, …, *s*_*n*_ that were triggered as on/off while performing activities instances *I*_*k*_*x* in a smart home and temporal information *Ti*. Each feature consists of total frequency, $F_{s}=\overset{I}{\underset{F_{s_{i}}}{\sum }}$, in the numeric form of the activated sensor during the progression of activity. The sensors not triggered while performing activities were assigned zero, $f_{kx}^{s}=0$. In this way, the feature matrix *F*_*k*_*x* consisting of activity instances *I*_*k*_*x* can be represented by the following Equation 1:


(1) $$\begin{array}{l} F_{kx}={\left\{f_{kx}^{s}\right\}}_{s=1}^{S} \end{array}$$

Since the values vary widely in ranges of raw data because healthy individuals and individuals with dementia performed the activities, there are high chances of abnormality in sensor frequency. In some machine learning algorithms, objective functions will not work correctly and efficiently. The feature matrix is shifted to a scaled version of a feature matrix to eliminate specific gross influences to address this problem. The *Rescaling method* has been used to normalize the range of features using Equation (2) as follows:


(2) $$\begin{array}{l} x^{\prime }=\frac{x-\text{min}\left(x\right)}{\text{max}\left(x\right)-\text{min}\left(x\right)} \end{array}$$

Scaling works better for ML models where the distance between the data points varies widely. In Equation (2), *x* is the real value of the instance, and *x*′ is the normalized value. The scaled feature matrix for all activities can be described by Equation (3), according to the proposed approach:


(3) $$\begin{array}{l} F_{kx}^{\prime }=\frac{f_{kx}^{s}-\text{min}\left(f_{kx}^{s}\right)}{\text{max}\left(f_{kx}^{s}\right)-\text{min}\left(f_{kx}^{s}\right)} \end{array}$$

Currently, the feature matrix is in shape to select the key feature. A set denoted by *S*_*k*_ is initially initialized with an empty set ϕ. Best key features *B*_*f*_ are extracted from activities *D*_*x*_ by counting the features of $\left\{f_{kx}^{s}\right\}$. In Equation (4), $P_{x}^{r}$ returns the number of features in a list containing all features $f_{kx}^{s}$ where each feature in $f_{kx}^{s}$ has a frequency greater than 0.


(4) $$\begin{array}{l} P_{x}^{r}=\left\{f_{kx}^{s}\right\}>0 \end{array}$$

(5) $$\begin{array}{l} {\overset{\;\sim }{P}}_{x}^{r}=P_{x}^{r}\geq U_{f} \end{array}$$

The value of *U*_*f*_ is user-defined, as shown in Equation (5), which allows the user to choose the number of best features from the feature matrix, and similarly, *U*_*i*_ in Equation (6) allows the user to select the number of instances. It provides full authority to the user to control the feature selection process, which could be sufficient for deciding the feature as a key feature to perform the health assessment of a smart home resident.


(6) $$\begin{array}{l} {\overset{\;\sim }{P}}_{x}^{r}=P_{x}^{r}\geq U_{i} \end{array}$$

The cross-validation technique is applied to assess the value of *U*_*i*_ and *U*_*f*_. The best-selected features are then appended to the empty set ϕ. If the selected feature is already in the set, it is discarded; else, it is appended. This process is repeated until each class's features are added or discarded.

The precedence is given to each feature *F*_*s*_ in a certain activity based on its occurrence. This process is repeated based on the maximum frequency in an activity to obtain an overall generic feature matrix. The features having low occurrence inactivity are discarded. Later, the feature matrix is formed based on Equation (6) for the best key features. The feature matrix features have maximum precedence in set ϕ as shown in Equation (7).


(7) $$\begin{array}{l} {\overset{-}{F}}_{kx}=\left\{f_{kx}^{s}\;argmax\left(p\right)\right\} \end{array}$$

### 3.5. Statistical key feature selection

Statistical features are a dataset's features which can be defined and calculated through statistical analysis. Statistical models are generic, increasing the capability of any model to recognize different activities at a fine-grained level. The common statistical features are bias, variance, mean, median, percentiles, standard deviation, etc. Researchers investigated that it is useful to use the statistical feature for human activity recognition (41). For example, it is proved that variance helps to achieve higher accuracy for different activities, such as walking, jogging, and hopping. The extracted statistical features are root mean square, standard deviation, mean, median, variance, averaged derivatives, zero-crossing rate, interquartile range, mean crossing rate, kurtosis, skewness, pairwise correlation, and spectral entropy from feature matrix generated by *PKFS*. After successfully extracting statistical features, these features are appended in the previous matrix made by *PKFS*. The statistical features are represented by _*S*_*f*_*kx*_, and the whole key feature matrix is represented by Equation (8).


(8) $$\begin{array}{l} {\overset{-}{F}}_{kx}=\left\{f_{kx}^{s}\;\epsilon \;argmax\left(p\right)\right\}\;\&\;{S_{f}}_{kx} \end{array}$$

### 3.6. XAI-HAR

Various traditional well-known feature selection techniques, namely, principal component analysis (PCA), minimum redundancy maximum relevance (mRMR), information gain (IG), and the proposed technique *XAI-HAR* is applied along with the machine learning algorithms random forest (RF), K-nearest neighbor (KNN), decision tree (C4.5), support vector machine (SVM), Heoffding tree (HT), multilayer perceptron (MLP), and naive Bayes (NB) for activity recognition. For further comparison, we also apply deep learning algorithms, such as deep neural network (DNN), convolution neural network (CNN), and CNN-based long short-term memory (CNN-LSTM). These methods for selecting feature selection are very effective in selecting the best features. These feature selection techniques are selected to compare and evaluate the proposed feature selection approach *XAI-HAR* for activity recognition. For PCA, we set the variance to 95%. We use local interpretable model agnostic (LIME) and apply it to RF (default parameters) to analyze the main components and explain essential features. LIME provides the model interpretability by producing meaningful and vital information. For KNN, the batch size is set to 100, the nearest neighbors are set to 1, the nearest neighbor searching algorithm is set to *LinearNNSearch*, and distance weighting is set to *False*. For the decision tree, the batch size is set to 100, the confidence factor is set to 0.25, *subtreeRaising* is set to *True*, and *reducErrorPruning* is set to *False*. For SVM, the batch size is set to 100, the complexity parameter is set to 1.0, the kernel is set to *PolyKernel*, and the tolerance parameter is set to 0.001. For NB, the batch size is set to 100, and *useKernalEstimator* is set to *false*. For DNN, the activation is *relu* in hidden layers and *softmax* in the output layer along with the optimizer as *adam*. For CNN and CNN-LSTM, the same parameters are set with the kernel_size as 3.

## 4. Experimental analysis and results

The proposed approach *XAI-HAR* is fundamentally different from other approaches in the way that *XAI-HAR* defines *K* subsets of features for *K* activity classes. In contrast, feature selection methods such as IG, mRMR, and PCA return a single subset of features from the existing feature set, given as input to selected classifiers. Furthermore, *XAI-HAR* uses the LIME-based RF model to analyze the main components and explain essential features. This section discusses the different valuation metrics for experimentation and evaluation. For experimentation, *CASAS-Cognitive Assessment Activity (Kyoto)* (27) dataset is used which is well known for cognitively impaired individuals research. Different experimental analyses are performed with different criteria on the dataset. Three-fold cross-validation (56) is applied for all experiments. This test leaves 1:3 part of the dataset for testing and 2:3 part for training. The KNN, SVM, DT C4.5, HT, MLP, and NB algorithms are used to evaluate the recognition results. For further comparison, we also apply deep learning algorithms, such as deep neural network (DNN), convolution neural network (CNN), and CNN-based long short-term memory (CNN-LSTM).

### 4.1. Evaluation metrics

The selection of evaluation metrics depends on the essence of the data. Accuracy is mainly considered a key evaluation metric when the data are balanced (i.e., an equal number of observations) (57). However, accuracy alone can be misleading if a dataset contains imbalanced observations in each category. To overcome this limitation, recall, precision, and f-score evaluation metrics are a rationale for the performance computation of *DFCII*. Given as under are the practical terms to help in evaluation and analysis. TP (i.e., true positive rate representing correctly categorized instances) calculates the accuracy by dividing it by N (all the samples of all activities). The recall measure is computed by TP divided by TP+FN (where FN is the false negative rate that provides wrongly recognized samples). We divide TP by TP+FP (false positive rate: samples of other activities wrongly recognized as one activity sample), and we obtain the precision of a technique. F-score shows the harmonic mean of recall and precision. The experiment's computing environment is set as Intel(R) Corei5, 8th Generation with 16 GB RAM, Windows 10 OS, and Python version 3.7.6 as shown in Table 2.


(9) $$\begin{array}{l} Accuracy=\frac{TP}{N} \end{array}$$

(10) $$\begin{array}{l} Recall=\frac{TP}{TP+FN} \end{array}$$

(11) $$\begin{array}{l} Precision=\frac{TP}{TP+FP} \end{array}$$

(12) $$\begin{array}{l} F-Score=2\times \;\frac{Precision\times Recall}{Precision+Recall} \end{array}$$

For cognitive health assessment, the f-score is used as a critical evaluation measure because the f-score is the most appropriate for the imbalanced data (57).

**Table 2:** Capability of the experimental machine.

**Type**	**Specification**
OS	Windows 10
CPU	Intel(R) Corei5, 8th Generation
RAM	16 GB
Python	3.7.6

**Figure 3:**
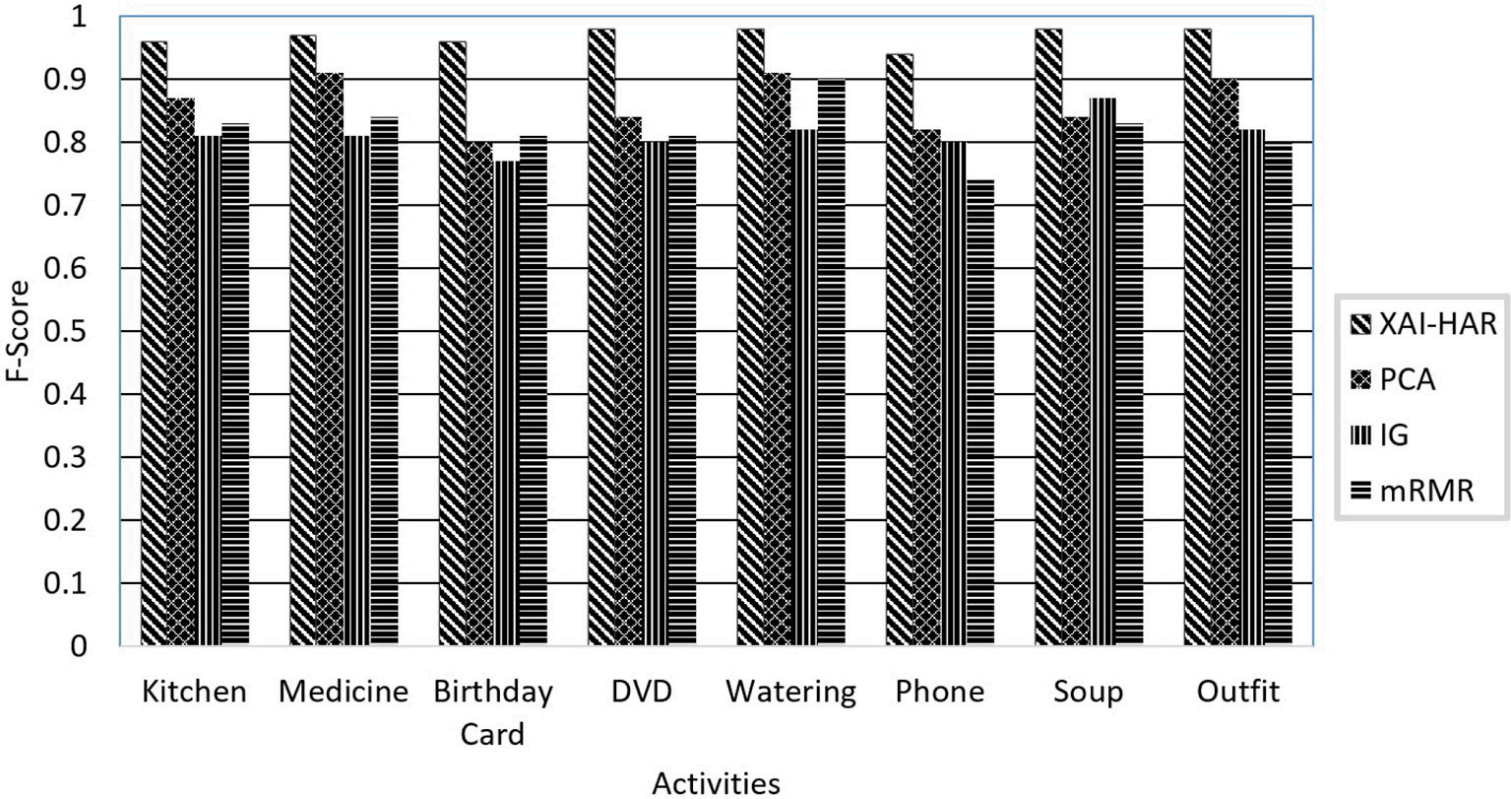
The comparison of the proposed *XAI-HAR* with feature selection methods such as PCA, mRMR, and IG in combination with RF for each activity of individuals with dementia and healthy individuals.

Figure 3 illustrates the f-score of each activity on activities of individuals with dementia and healthy individuals when *XAI-HAR*, PCA, IG, and mRMR are applied with the RF learning method. For kitchen activity, the *XAI-HAR* achieves 96% f-score, while PCA, IG, and mRMR achieve 87, 81, and 83% f-score, respectively. For medicine activity, the *XAI-HAR* achieves 97% f-score, while PCA, IG, and mRMR achieve 91, 81, and 84% f-score, respectively. For birthday card activity, the *XAI-HAR* achieves 94% f-score, while PCA, IG, and mRMR achieve 80, 77, and 81% f-score, respectively. In the case of DVD activity, the *XAI-HAR* achieves 98% f-score, while PCA, IG, and mRMR achieve 84, 80, and 81% f-score, respectively. For watering activity, the *XAI-HAR*, PCA, IG, and mRMR achieve 98, 91, 82, and 90% f-score, respectively. For phone activity, the *XAI-HAR*, PCA, IG, and mRMR achieve 94, 82, 80, and 74% f-score, respectively. In the case of soup activity, the *XAI-HAR* achieves 98% f-score, while PCA, IG, and mRMR achieve 84, 87, and 83% f-score, respectively. For outfit activity, the *XAI-HAR* achieves 98% f-score, while PCA, IG, and mRMR achieve 90, 82, and 80% f-score, respectively. It is seen that all feature selection methods achieved less accurate results than *XAI-HAR* when the activities performed by individuals with dementia and healthy individuals were classified collectively.

**Figure 4:**
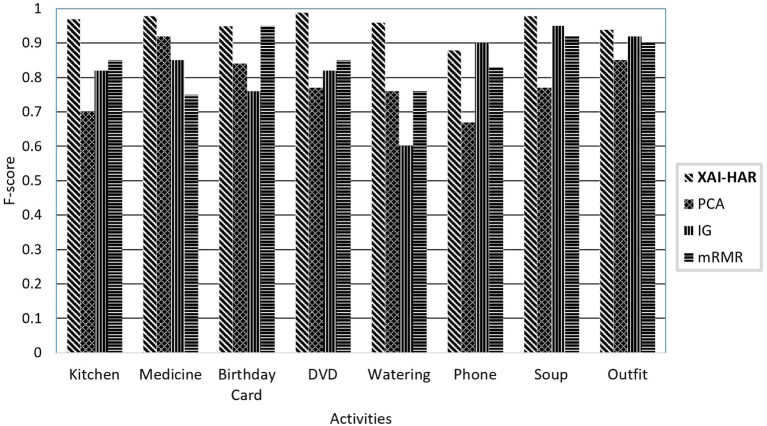
The comparison of the proposed *XAI-HAR* with feature selection methods such as PCA, mRMR, and IG in combination with RF for each activity of healthy individuals.

Figure 4 presents the f-score of each activity on the healthy individual's activities when *XAI-HAR*, PCA, IG, and mRMR are applied with the RF learning method. For kitchen activity, the *XAI-HAR* achieves 96% f-score, while PCA, IG, and mRMR achieve 70, 82, and 85% f-score, respectively. For medicine activity, the *XAI-HAR* achieves 98% f-score, while PCA, IG, and mRMR achieve 92, 85, and 75% f-score, respectively. For birthday card activity, the *XAI-HAR* achieves 95% f-score, while PCA, IG, and mRMR achieve 84, 76, and 95% f-score, respectively. In the case of DVD activity, the *XAI-HAR* achieves 99% f-score, while PCA, IG, and mRMR achieve 77, 82, and 85% f-score, respectively. For watering activity, the *XAI-HAR*, PCA, IG, and mRMR achieve 96, 76, 60, and 76% f-score, respectively. For phone activity, the *XAI-HAR*, PCA, IG and mRMR achieve 93, 67, 90, and 83% f-score, respectively. In the case of soup activity, the *XAI-HAR* achieves 98% f-score, while PCA, IG, and mRMR achieve 77, 95, and 92% f-score, respectively. For outfit activity, the *XAI-HAR* achieves 95% f-score, while PCA, IG, and mRMR achieve 85, 92, and 90% f-score, respectively. The results conclude that all feature selection methods achieve less accuracy than *XAI-HAR* when only the activities performed by healthy individuals are classified.

**Figure 5:**
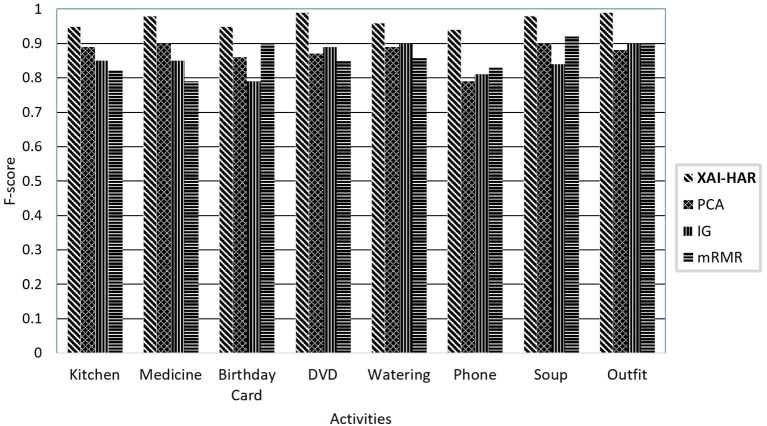
Comparison of proposed *XAI-HAR* with feature selection methods such as PCA, mRMR, and IG in combination with RF for each activity of individuals with dementia.

Figure 5 presents the f-score of each activity on the activities of individuals with dementia when *XAI-HAR*, PCA, IG, and mRMR are applied with the RF learning method. For kitchen activity, the *XAI-HAR* achieves 95% f-score, while PCA, IG, and mRMR achieve 89, 85, and 82% f-score, respectively. For medicine activity, the *XAI-HAR* achieves 98% f-score, while PCA, IG, and mRMR achieve 90, 85, and 79% f-score, respectively. For birthday card activity, the *XAI-HAR* achieves 96% f-score, while PCA, IG, and mRMR achieve 86, 79, and 90% f-score, respectively. In the case of DVD activity, the *XAI-HAR* achieves 99% f-score, while PCA, IG, and mRMR achieve 87, 89, and 85% f-score, respectively. For watering activity, the *XAI-HAR*, PCA, IG, and mRMR achieve 96, 89, 90, and 86% f-score, respectively. For phone activity, the *XAI-HAR*, PCA, IG, and mRMR achieve 94, 79, 81, and 83% f-score, respectively. In the case of soup activity, the *XAI-HAR* achieves 98% f-score, while PCA, IG, and mRMR achieve 90, 84, and 92% f-score, respectively. For outfit activity, the *XAI-HAR* achieves 99% f-score, while PCA, IG, and mRMR achieve 88, 90, and 90% f-score, respectively. It is shown that all feature selection methods achieved less accurate results than XAI-HAR when only the activities performed by individuals with dementia are classified.

Table 3 presents a comparison of *XAI-HAR* with PCA, IG, and mRMR using the performance evaluation metrics on the CASAS dataset (27). We use KNN, SVM, DT, NB, HT, MLP, RF, DNN, CNN, and CNN-LSTM learning models for comparison. *XAI-HAR* improves recognition performance compared with all other models. *XAI-HAR* achieves the best accuracy of 96.4% in combination with RF compared with KNN, SVM, DT, HT, MLP, and NB. While analyzing the *XAI-HAR* with existing feature selection approaches, i.e., PCA, IG, and mRMR, *XAI-HAR* achieves better results. The *XAI-HAR* with RF achieved a 5% high f-score compared with PCA-based learning models on activities of healthy individuals and individuals with dementia. Similarly, *XAI-HAR* with RF achieved a 5% high f-score compared with IG-based learning models on activities of healthy individuals and individuals with dementia. The *XAI-HAR* with RF achieved a 12% high f-score compared with mRMR-based learning models on activities of healthy individuals and individuals with dementia While on activities of individuals with dementia, *XAI-HAR* with RF achieved a 13% high f-score compared with PCA-based learning models. Similarly, *XAI-HAR* with RF achieved an 11% high f-score compared with IG-based learning models on activities of healthy individuals and individuals with dementia. *XAI-HAR* with RF achieved a 3% high f-score compared with mRMR-based learning models on activities of healthy individuals and individuals with dementia. Finally, in healthy individuals' activities, *XAI-HAR* with RF achieved a 6% high f-score compared with PCA-based learning models. Similarly, *XAI-HAR* with RF achieved a 9% high f-score compared with IG-based learning models on activities of healthy individuals and individuals with dementia. *XAI-HAR* with RF achieved an 8% high f-score compared with mRMR-based learning models on activities of healthy individuals and individuals with dementia.

**Table 3:** F-score-based comparison of *XAI-HAR* with other feature selection and classification techniques for activities in CASAS Dataset (27).

**Approach**	**Participants**	**K-NN**	**SVM**	**DT**	**NB**	**RF**	**MLP**	**HT**	**DNN**	**CNN**	**CNN-LSTM**
XAI-HAR	Healthy & Dementia	0.96	0.92	0.90	0.89	**0.96**	0.83	0.75	0.95	0.95	0.95
PCA	Healthy & Dementia	0.86	0.87	0.87	0.89	0.91	0.82	0.75	0.90	0.81	0.81
IG	Healthy & Dementia	0.81	0.83	0.82	0.80	0.91	0.96	0.84	0.96	0.95	0.96
mRMR	Healthy & Dementia	0.82	0.82	0.84	0.83	0.84	0.83	0.75	0.95	0.95	0.95
XAI-HAR	Dementia	0.94	0.89	0.86	0.85	**0.95**	0.89	0.62	0.77	0.80	0.80
PCA	Dementia	0.79	0.82	0.79	0.80	0.82	0.88	0.78	0.91	0.87	0.87
IG	Dementia	0.83	0.83	0.82	0.81	0.84	0.88	0.78	0.91	0.88	0.88
mRMR	Dementia	0.85	0.86	0.84	0.84	0.92	0.89	0.76	0.97	0.86	0.86
XAI-HAR	Healthy	0.95	0.91	0.89	0.88	**0.97**	0.96	0.79	0.96	0.95	0.95
PCA	Healthy	0.87	0.86	0.87	0.85	0.91	0.96	0.79	0.96	0.96	0.96
IG	Healthy	0.85	0.84	0.83	0.82	0.88	0.96	0.83	0.96	0.96	0.96
mRMR	Healthy	0.86	0.87	0.85	0.81	0.89	0.95	0.86	0.96	0.94	0.94

Key: HT-heofffding tree. The bold values indicate the superior results.

It is noticed in most cases that the RF outperforms all other classifiers in terms of accuracy, and the HT classifier has the lowest accuracy.

Table 4 presents the time complexity of all models. Experiments reveal that the least model compiling time of the XAI-HAR approach on healthy and dementia individuals dataset is 0.01 s using KNN, and the highest model compiling time is 246 s using the CNN-LSTM model. Next, the least model compiling time of the PCA feature selection approach on the healthy and dementia individuals dataset is 0.11 s using NB, and the highest model compiling time is 195 s using the MLP classifier. Furthermore, the least model compiling time of the IG feature selection approach on healthy and dementia individuals dataset is 0.01 s using KNN, and the highest model compiling time is 55 s using the CNN-LSTM classifier. Furthermore, the least model compiling time of the mRMR feature selection approach on the healthy and dementia individuals dataset is 0.01 s using KNN, and the highest model compiling time is 324 s using the CNN-LSTM classifier. Next, the least model compiling time of the XAI-HAR approach on the dementia individuals dataset is 0.01 s using KNN, and the highest model compiling time is 21 s using the CNN-LSTM model. Furthermore, the least model compiling time of the PCA feature selection approach on the dementia individuals dataset is 0.01 s using KNN, and the highest model compiling time is 24 s using the CNN-LSTM model. Furthermore, the least model compiling time of the IG feature selection approach on the dementia individuals dataset is 0.01 s using KNN, and the highest model compiling time is 10 s using the CNN-LSTM model. Furthermore, the least model compiling time of the mRMR feature selection approach on the dementia individuals dataset is 0.01 s using KNN, and the highest model compiling time is 3 s using the CNN-LSTM model. Next, the least model compiling time of the XAI-HAR approach on the healthy individuals dataset is 0.01 s using KNN, and the highest model compiling time is 144 s using the CNN-LSTM model. Furthermore, the least model compiling time of the PCA feature selection approach on the healthy individuals dataset is 0.01 s using KNN, and the highest model compiling time is 144 s using the CNN-LSTM model. Furthermore, the lowest model compiling time of the IG feature selection approach on the healthy individuals dataset is 0.01 s using KNN, and the highest model compiling time is 82 s using the CNN model. Furthermore, the least model compiling time of the mRMR feature selection approach on the healthy individuals dataset is 0.01 s using KNN, and the highest model compiling time is 42 s using the CNN model.

**Table 4:** Time-based comparison of *XAI-HAR* with other feature selection and classification techniques for activities in the casas dataset (27).

**Approach**	**Participants**	**K-NN**	**SVM**	**DT**	**NB**	**RF**	**MLP**	**HT**	**DNN**	**CNN**	**CNN-LSTM**
XAI-HAR	Healthy & Dementia	0.01	0.41	0.59	0.1	**2.5**	194	0.7	42	77	246
PCA	Healthy & Dementia	0.8	0.44	0.49	0.11	2.6	195	0.55	5	9	26
IG	Healthy & Dementia	0.01	0.2	0.2	0.03	1.37	11.6	0.09	10.9	24	55
mRMR	Healthy & Dementia	0.01	0.39	0.59	0.11	2.72	2.41	0.07	12	89	324
XAI-HAR	Dementia	0.01	0.14	0.01	0.01	**0.2**	5.41	0.01	3	5	21
PCA	Dementia	0.01	0.07	0.01	0.01	0.2	5.21	0.02	3	5	24
IG	Dementia	0.01	0.25	0.01	0.01	0.13	1.41	0.01	2.9	7.0	10.4
mRMR	Dementia	0.01	0.1	0.01	0.01	0.12	0.41	0.01	3	3	3
XAI-HAR	Healthy	0.01	0.21	0.27	0.06	**1.61**	56	0.37	21	61	144
PCA	Healthy	0.01	0.24	0.36	0.05	1.78	56.2	0.2	12	45	144
IG	Healthy	0.01	0.3	0.15	0.03	1.17	14.2	0.1	20.5	82.0	77.0
mRMR	Healthy	0.01	0.24	0.18	0.02	1.45	5.33	0.05	15	42	36

The bold values indicate the superior results.

Table 5 presents the confusion matrix of the proposed approach. It shows how many instances of one activity get confused with instances of other activities. The kitchen activity is getting confused with the phone activity. The birthday card activity and phone activity are getting confused with each other. In comparison, the remaining five activities are recognized accurately. Overall, *XAI-HAR* achieved better results than other approaches.

**Table 5:** Confusion matrix of *XAI-HAR* for activities in the CASAS dataset (27).

**Activities**	**Kit**	**Med**	**BC**	**DVD**	**Wat**	**Phone**	**Soup**	**Outfit**
Kit	321	0	0	0	1	14	1	1
Med	0	329	2	0	0	0	4	0
BC	0	0	311	0	0	24	0	0
DVD	0	0	1	328	0	1	0	1
Wat	5	0	0	0	335	0	0	0
Phone	3	1	24	3	0	272	0	4
Soup	1	6	0	0	0	0	326	0
Outfit	0	0	1	0	0	0	0	342

Kit, Kitchen; Med, Medicines; Wat, Watering; BC, Birthday Card.

### 4.2. Explainable RF with local interpretable model agnostic for healthy individuals

We use local interpretable model agnostic (LIME) and apply it to RF to analyze the main components and explain essential features. LIME provides the model interpretability by producing meaningful and vital information. We also use ELI5 to inspect machine learning classifiers and explain their predictions. ELI5 extracts the top 10 features with their corresponding weights.

#### 4.2.1. Interpretation of healthy individuals and individuals with dementia

**Figure 6:**
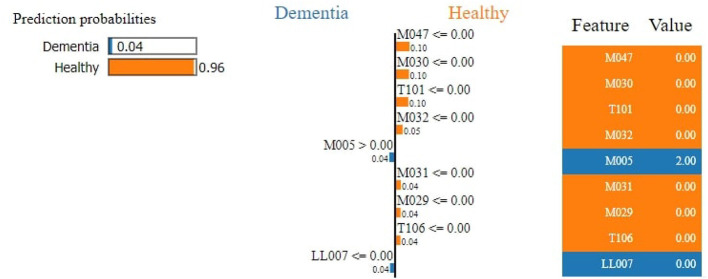
Feature explainability.

The RF model achieves an accuracy score of 96.25%. The result of the LIME model gives a list of essential features and explains each feature's contribution to the dataset's prediction. Figure 6 shows the output of the LIME model and explains the top 10 features. The leftmost sections present the prediction probabilities with 0.96% healthy and 0.04% dementia probability values. The second section represents the 10 most important features. We use binary classification and that is why it is in two colors, blue and orange. Attributes in orange color support the healthy class, and the blue color supports the dementia class. Floating-point numbers on the horizontal bar show the importance of the features. M047, M030, and T101 are the Top three features belonging to the healthy class. The rightmost section contains the actual values of the top 10 variables.

**Figure 7:**
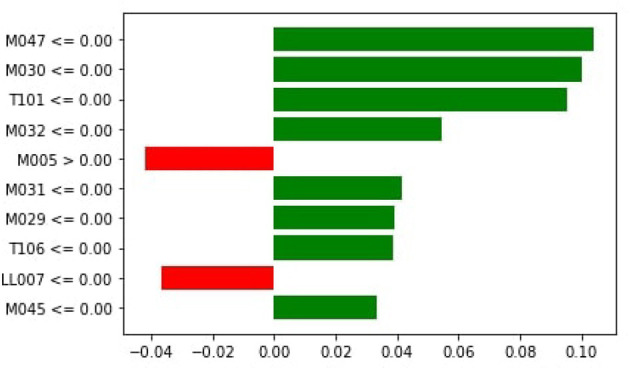
Local importance.

**Figure 8:**
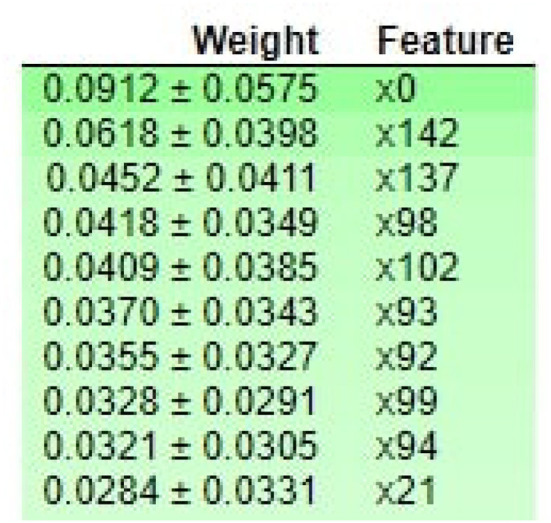
ELI5-based feature Inspection.

Figure 7 demonstrates that M047, M030, T101, MO32, and M031 are the top most important features of the model belonging to the healthy class. While M005 and LL007 are the top most important features of the model belonging to the dementia class, Figure 8 provides the weights against to top 10 features participating most in the prediction process.

#### 4.2.2. Interpretation of healthy individuals

**Figure 9:**
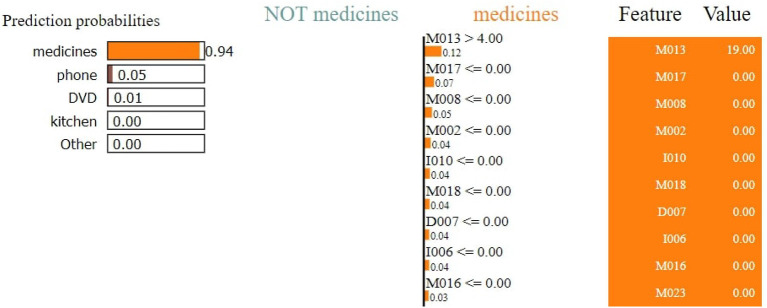
Feature explainability.

The RF achieves an accuracy score of 97.40%. The result of the LIME model gives a list of essential features and explains each feature's contribution to the dataset's prediction. Figure 9 shows the output of the LIME model and explains the top 10 features. The leftmost sections present the prediction probabilities with 0.94% for medicine and 0.05% for phone probability values. For medicine, it is noticed that M013, M017, M017, MO08, M002, I010, M018, D007, I006, and M016 are the top most important features of the model belonging to the healthy class.

The second section represents the 10 most important features. Attributes in orange color support the medicine class and the purple color supports the phone class. Floating-point numbers on the horizontal bar show the importance of the features. M013, M017, and M008 are the top three features belonging to the medicine class. The rightmost section contains the actual values of the top 10 variables.

**Figure 10:**
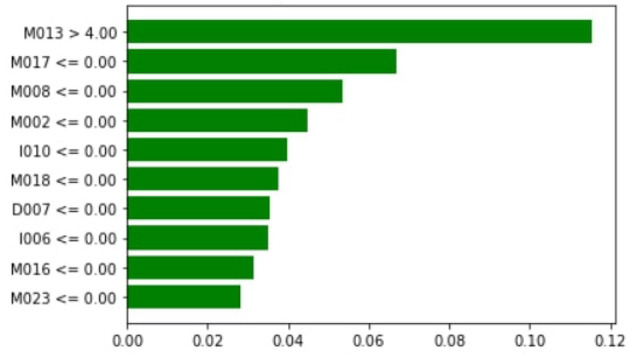
Local importance.

**Figure 11:**
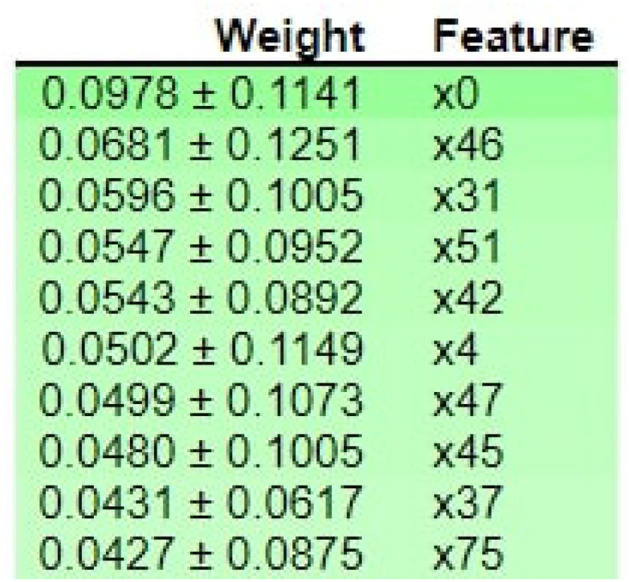
ELI5-based feature Inspection.

Figure 10 shows that M013, M017, M008, M051, I006, I010, M023, M015, and D007 are the top three most essential features of the model belonging to the medicine class. Figure 11 provides the weights against to top 10 features participating most in the prediction process.

#### 4.2.3. Interpretation of individuals with dementia

**Figure 12:**
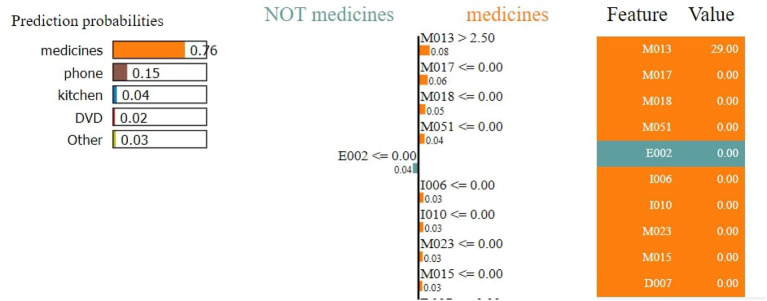
Feature explainability.

The RF model achieves an accuracy score of 93.93%. The result of the LIME model gives a list of essential features and explains each feature's contribution to the dataset's prediction. Figure 12 shows the output of the LIME model and explains the top 10 features. The leftmost sections present the prediction probabilities with 0.76% for the medicine class, 0.15% for the phone class, 0.04 for kitchen, 0.02 for DVD, and 0.03 for other probability values. The second section represents the 10 most important features–attributes in orange color support the medicine class and others support not medicine class. Floating-point numbers on the horizontal bar show the importance of the features. M013, M017, and M018 are the Top three features belonging to the healthy class. The rightmost section contains the actual values of the top 10 variables.

**Figure 13:**
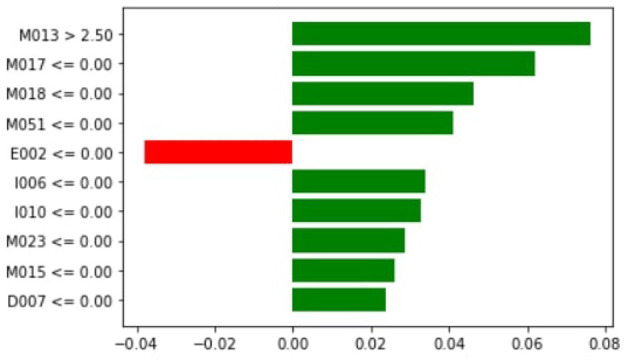
Local importance.

**Figure 14:**
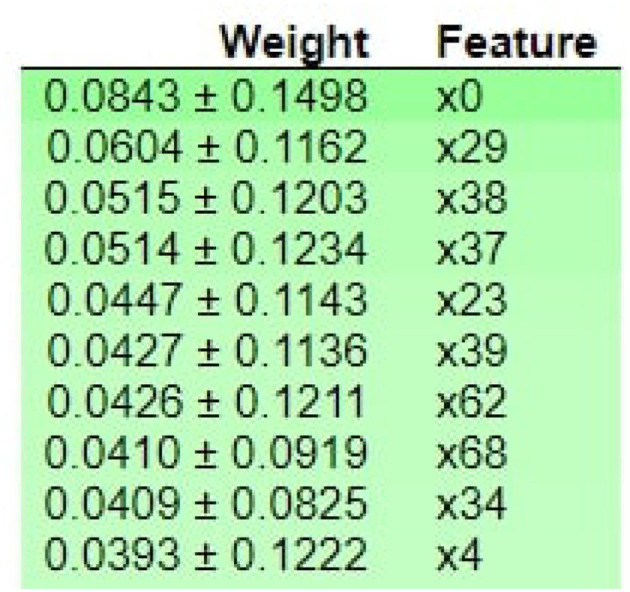
ELI5-based feature Inspection.

Figure 13 shows that M013, M017, and M018 are the top three most essential features of the model belonging to the healthy class. Figure 14 provides the weights against to top 10 features participating most in the prediction process.

### 4.3. Discussion

Currently, clinicians are interested in insight into an individual's functional ability to detect diseases early. This study presented an XAI-empowered human activity recognition approach for individuals with dementia and healthy individuals to monitor their health. RF achieves the best results by using the XAI-HAR feature matrix. The other learning models, such as KNN, SVM, HT, MLP, NB, DNN, CNN, and CNN-LSTM, achieve better f-score using XAI-HAR-based feature matrix than PCA, IG, and mRMR-based feature matrix. However, DT showed relative degradation in dementia individuals' activities compared with others. The rationale behind this degradation is due to the fact that the data collected for dementia individuals have non-normal distribution. In addition, the number of dementia individuals performing activities is also fewer than that of healthy individuals. The KNN looks for the nearest neighbors in the dementia individual's activities for assigning labels. The SVM looks for the boundaries of the target variable in the dataset's search space for assigning labels.

In contrast, DT looks for promising interactions between features representing activities of an individual with dementia. We also provide the explainability of the prediction made by the RF model. We use local interpretable model agnostic (LIME) and apply it to RF to analyze the main components and explain the most important features. LIME provides the model interpretability by producing meaningful and vital information. We use ELI5 to inspect machine learning classifiers and explain their predictions. ELI5 extracts the top 10 essential features with their corresponding weights. As shown in Table 3, it is noticed that the RF achieves a 2% higher f-score than the deep learning models such as DNN, CNN, and CNN-LSTM while using XAI-HAR-based feature matrix. In addition, the deep learning models take more time in model building than RF, as shown in Table 4. The deep learning models not only achieve better f-score on PCA, IG, and mRMR-based feature matrices than RF but also take a long time in model training. So, the RF model works more robustly and efficiently on the XAI-HAR feature matrix than all other learning models. Below, we answer the research questions articulated in this study.

**Answer to RQ1:** The *XAI-HAR* consists of two steps: *physical key features selection (PKFS)* and *statistical key features selection (SKFS)* to form a feature matrix corresponding to different well-established contemporary methods used for recognizing activities. Further, we use local interpretable model agnostic (LIME) to interpret the decision-making process by classifiers. **Answer to RQ2:** *XAI-HAR* presents the concept of selecting local key features within the dataset while maintaining the original meaning of the features.**Answer to RQ3:** The weighting criteria are set as explained in equations 1, 2, 3, 4, 5, 6, 7, and 8.**Answer to RQ4:** The results reveal that the proposed approach will help neuropsychologists and clinicians to gain insight into an individual's functional ability to detect diseases and recognize their daily activities. Furthermore, the proposed approach help to understand the reason behind decision-making since detecting cognitive impairment is critical. Finally, it helps to provide interpretability to individuals with dementia.

## 5. Conclusion and future work

This study presented an XAI-empowered human activity recognition approach to enhance the recognition accuracy of cognitively impaired individuals' activities in a smart home. This approach helps to monitor the activities of cognitively impaired individuals and individuals having chronic impairments. The proposed approach improved the recognition accuracy of the intra-class variations. Moreover, *XAI-HAR* is compared with other commonly used feature selection techniques (PCA, mRMR, and IG) from the literature and other machine learning techniques. The results showed that the *XAI-HAR* achieved an f-score of 96% using RF, which is higher than other feature selection approaches. In addition, these results demonstrated the further help provided by the proposed *XAI-HAR* to achieve healthier patients over available patients. In future, we aim to experiment with the proposed approach on the dataset having complex activities. We also intend to develop a dataset with multiple participants of different ages and pets. It will be challenging to detect activities and cognitive conditions in the presence of pets. Furthermore, we intend to extend this study by providing essential features and early detection for other domains, particularly for Parkinson's and Alzheimer's diseases.

## Data availability statement

The original contributions presented in the study are included in the article/Supplementary material, further inquiries can be directed to the corresponding authors.

## Author contributions

All authors listed have made a substantial, direct, and intellectual contribution to the work and approved it for publication.

## Footnotes

^1^ http://casas.wsu.edu/datasets/
